# Notice of Wood’s Practice of Medicine

**Published:** 1852-07

**Authors:** 


### EDITORIAL.

*A Treatise on the Practice of Medicine.* By Geo. B. Wood, M.D., Prof. of the Theory and Practice of Medicine in the University of Penn.; President of the College of Physicians of Phila.; one of the Physicians of the Penn. Hospital; one of the Authors of the U. S. Dispensatory, etc., etc. Third edition. In two vol., octavo. Phil.: Lippincot, Grambo & Co.; 1852. From the publishers.

The fact that another edition of this work has been so soon demanded, is evidence that it is appreciated by the profession.

The following extract from the preface to the third edition will show the improvements that have been made in the matter of the work:

"In all its essential features, the treatise remains unchanged; but the multiplicity of observation, and the progress of discovery, within the last few years, have been such as to render many alterations and additions and some elisions necessary, in order to adapt it to the present state of medical knowledge. The reader will find evidences of such modifications scattered here and there throughout the work. Among the additions may be mentioned brief notices of several diseases, which from their rarity, or total absence in this country, or from being yet unknown as distinct affections, were not described in the former editions. Such are the relapsing fever of Dr. Jenner, the leucocythemia of Professor Bennet, the dengue, and certain cutaneous affections, as trichosis, lupus, and pellagra. The chief modifications, however, have reference to subjects previously more or less fully discussed, as, for example, to inflammation, fatty degeneration, carcinoma, epidemic cholera, the treatment of phthisis, the nature of hemorrhage, Bright's disease, &c. The reader who may deem it worth while to compare the present with former editions, will probably also notice, among the most prominent changes, a more extended reference to the important microscopic discoveries of recent times, and their bearing upon pathology."

In our opinion, Dr. Wood as a medical writer has few equals either in clearness and beauty of style, or in the happy manner in which he applies science and philosophy to the art of curing. His book is an honor to American physicians, and we recommend it to our readers, assuring them that if they experience as much pleasure as we have ourselves in its perusal, they will be amply repaid.

